# *Saccharomyces boulardii* CNCM I-745 supplementation reduces gastrointestinal dysfunction in an animal model of IBS

**DOI:** 10.1371/journal.pone.0181863

**Published:** 2017-07-21

**Authors:** Paola Brun, Melania Scarpa, Chiara Marchiori, Gloria Sarasin, Valentina Caputi, Andrea Porzionato, Maria Cecilia Giron, Giorgio Palù, Ignazio Castagliuolo

**Affiliations:** 1 Department of Molecular Medicine, University of Padova, Padova, Italy; 2 Department of Neurosciences, University of Padova, Padova, Italy; 3 Department of Pharmacological Sciences, University of Padova, Padova, Italy; University of Nevada School of Medicine, UNITED STATES

## Abstract

**Background:**

We evaluated the effect of *Saccharomyces boulardii* CNCM I-745 on intestinal neuromuscular anomalies in an IBS-type mouse model of gastrointestinal motor dysfunctions elicited by *Herpes Simplex* Virus type 1 (HSV-1) exposure.

**Methods:**

Mice were inoculated intranasally with HSV-1 (10^2^ PFU) or vehicle at time 0 and 4 weeks later by the intragastric (IG) route (10^8^ PFU). Six weeks after IG inoculum, mice were randomly allocated to receive oral gavage with either *S*. *boulardii* (10^7^ CFU/day) or vehicle. After 4 weeks the following were determined: a) intestinal motility using fluorescein-isothiocyanate dextran distribution in the gut, fecal pellet expulsion, stool water content, and distal colonic transit of glass beads; b) integrity of the enteric nervous system (ENS) by immunohistochemistry on ileal whole-mount preparations and western blot of protein lysates from ileal longitudinal muscle and myenteric plexus; c) isometric muscle tension with electric field and pharmacological (carbachol) stimulation of ileal segments; and d) intestinal inflammation by levels of tumor necrosis factor α, interleukin(IL)-1β, IL-10 and IL-4.

**Results:**

*S*. *boulardii* CNCM I-745 improved HSV-1 induced intestinal dysmotility and alteration of intestinal transit observed ten weeks after IG inoculum of the virus. Also, the probiotic yeast ameliorated the structural alterations of the ENS induced by HSV-1 (i.e., reduced peripherin immunoreactivity and expression, increased glial S100β protein immunoreactivity and neuronal nitric oxide synthase level, reduced substance P-positive fibers). Moreover, *S*. *boulardii* CNCM I-745 diminished the production of HSV-1 associated pro-inflammatory cytokines in the myenteric plexus and increased levels of anti-inflammatory interleukins.

**Conclusions:**

*S*. *boulardii* CNCM I-745 ameliorated gastrointestinal neuromuscular anomalies in a mouse model of gut dysfunctions typically observed with irritable bowel syndrome.

## Introduction

Irritable bowel syndrome (IBS) is a functional gastrointestinal disorder that significantly affects patient quality of life [1,2]. It is the most commonly diagnosed gastrointestinal condition affecting up to 10−15% of the population in industrialized countries [2,3]. IBS covers a range of polymorphous clinical manifestations, such as abdominal distension, pain or discomfort, and changes in bowel habits with a chronic irregular course [2]. Based on the prevalent symptoms three types of IBS are described: diarrheal, constipation and alternating forms. Visceral hypersensitivity, gastrointestinal dysmotility, post infectious reactivity, brain-gut interactions, microscopic inflammation, and gut dysbiosis, have been implicated in the pathogenesis of IBS and demonstrate the challenge that IBS still represents for clinicians and researchers.

The etiology of IBS has not been completely established to date. Neuroplasticity disorders of the enteric nervous system (ENS) have been described in functional and inflammatory bowel diseases [4]. Although the underlying process remain elusive, immune-inflammatory mechanisms have been hypothesized [5]. Infiltrating CD3 lymphocytes have been detected in the myenteric plexus of patients with severe gastrointestinal motor disorders requiring surgical resection, such as gastroparesis, chronic intestinal pseudo-obstruction, and slow-transit constipation [6]. Abnormal association between mast cells and nerve fibers, and increased release of tryptase and histamine have also been described in IBS patients [7], suggesting that histamine and other bioactive substances released from mast cells infiltrating the gut mucosa could affect the behavior of nearby enteric nerves [8].

The limited knowledge of the cellular and molecular mechanisms underlying IBS combined with the lack of reliable animal models that reproduce all aspects of this debilitating disease have hampered the search for effective treatments. Our research group has established an animal model of persistent infection with *Herpes simplex* virus type 1 (HSV-1) in the ENS [9]. The persistent viral infection of the ENS leads to gastrointestinal neuromuscular structural and functional anomalies (i.e. gut dysmotility followed by periods of regular GI transit) and to a slight mucosal inflammation mimicking, at least in part, those described in IBS [9].

Probiotics are an attractive non-pharmacological option for the treatment of IBS since these live microorganisms exert beneficial effects on gut homeostasis [10]. They suppress the growth and binding of pathogenic bacteria, improve the barrier function of the intestinal epithelium, and tune the immune activity of the host [10,11,12], either by direct interaction with immune cells or by production of by-products of fiber fermentation [13]. Moreover, probiotics have been shown to improve bowel dysmotility [14]. Among probiotics, *Saccharomyces boulardii* CNCM I-745 is a non-pathogenic yeast widely used in the prophylaxis and treatment of diarrheal diseases [15]. It enhances gastrointestinal barrier function and strongly modulates mucosal inflammatory and immune responses [15]. Since it has been reported that *S*. *boulardii* CNCM I-745 supplementation significantly improves the quality of life of IBS patients [16], we designed this study to investigate the effects of this probiotic yeast supplementation in our IBS-type murine model of HSV-1 induced gut dysmotility.

## Materials and methods

### Animals

Six-week-old C57BL/6J male mice, weighing 18±1.3 g, were purchased from Envigo Laboratories (Oderzo, Italy) and housed in groups of 4 per cage, in a temperature-controlled environment (22 ± 2°C) under a 12-hour light-dark cycle. Standard mouse chow food and water were provided *ad libitum*. All experimental protocols were approved by the Animal Care and Use Ethics Committee of the University of Padova under license from the Italian Ministry of Health, and they were in compliance with the National and European guidelines for handling and use of experimental animals.

### HSV-1 strains

HSV-1 strain 16 was propagated in Vero cells (American Type Culture Collection, Manassas, VA). The viral titer was quantified using the plaque assay. Vero cells grown in 12-well plates (2x10^5^ cells per well) were infected with 10-fold serial dilutions of virus. After 2 hours, monolayers were then covered with nutrient medium supplemented with 2% sterile agar to prevent the virus spreading to nearby uninfected cells. Plates were incubated for additional 46 hours and plaques produced at particular dilution were counted to provide the number of infectious virions as means plaque-forming units (PFU) per milliliter [9]. Viral stocks were aliquoted and stored at −80°C. Each aliquot was used in a single experiment.

### *S*. *boulardii* preparation

Lyophilized *S*. *boulardii* CNCM I-745 (*Sb*, Biocodex, France) was re-suspended in sterile phosphate buffered saline (PBS) and left at room temperature for 10 minutes. The titer of the solution was periodically checked on Sabouraud agar plates. *S*. *boulardii* CNCM I-745 (10^7^ CFU/mouse) was administered daily by oral gavage in a total volume of 100 μL. Sterile PBS was used as vehicle.

### Mice infection and treatments

**Fig 1:**
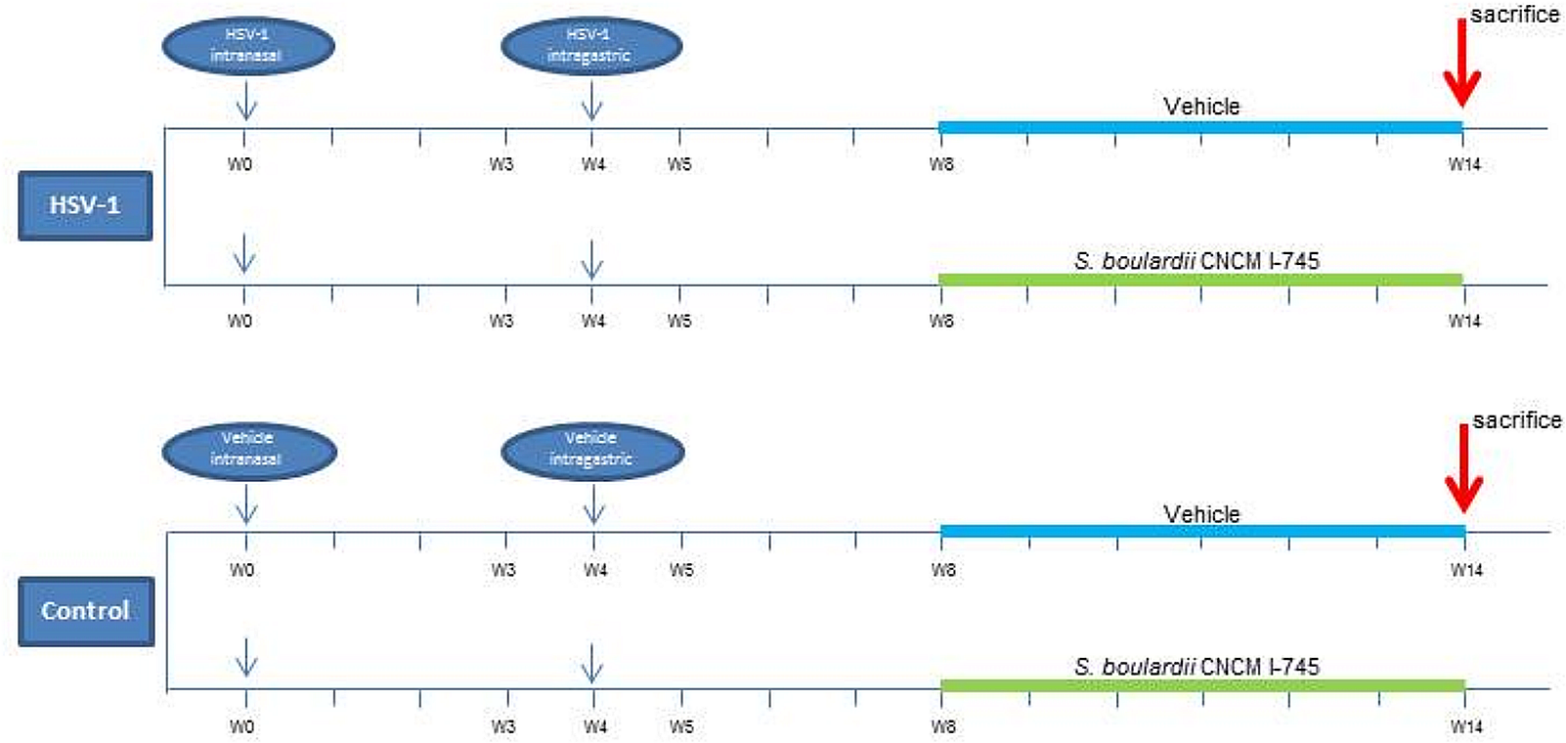
Scheme for the experimental course of the *S*. *boulardii* CNCM I-745 supplementation in HSV-1 infected mice. Mice were intranasally (IN) inoculated with Vero cell lysate (vehicle) or HSV-1 (10^2^ PFU). After 4 weeks, mice received an intragastric inoculum (IG) of Vero cell lysate (vehicle) or HSV-1 (10^8^ PFU). Mice were randomly allocated to receive 100 μL of either phosphate buffered saline (vehicle) or *S*. *boulardii* CNCM I-745 by daily oral gavage starting 4 weeks after IG viral inoculum. Animals were sacrificed 10 weeks after IG viral inoculum. n = 8 mice for each experimental group.

After a 1-week acclimatization period, mice were randomly assigned to two groups (HSV-1 infected and controls), and further divided into two subgroups as shown in Fig 1. Mice were infected by intranasal instillation of HSV-1 (10^2^ PFU/mouse) in a total volume of 10 μL, or received an equal volume of vehicle (controls). Mice were then monitored daily for the emergence of any neurological deficit. Four weeks later, the infected animals were inoculated again with HSV-1 (100 μL; 10^8^ PFU/mouse) by intragastric (IG) gavage (24 gauge, 9-cm catheter). Controls received equal volumes of Vero cell lysate.

Besides randomization between HSV-1 infection and control, mice were randomly assigned to receive either *S*. *boulardii* CNCM I-745 supplementation or placebo daily (*Sb*-supplemented or vehicle-treated mice; Fig 1). *S*. *boulardii* CNCM I-745 supplementation started 4 weeks after IG vehicle/viral inoculum and continued for 6 weeks. Food intake and body weight changes were recorded weekly throughout the experiment. Animals were sacrificed 10 weeks after IG viral inoculum by cervical dislocation [17] (Fig 1).

### Neurological assessment

Starting with IN viral inoculum neurological integrity of each mouse was assessed at weekly interval by a blinded observer using a validated scoring system [18]. Six tests were performed to evaluate sensorimotor functions: (1) spontaneous activity; (2) symmetry in the movement of the four limbs; (3) forepaw outstretching; (4) climbing; (5) body proprioception; and (6) response to vibrissae touch.

### Histopathological evaluation

Ileal (5−10 cm from ileocecal valve) and colonic (1−5 cm from the cecum) specimens were fixed in formalin and paraffin embedded. Sections (5 μm thick) were stained with hematoxylin/eosin. Slides (6−8/mouse) were assessed in a blinded manner. A minimum of 10 independent fields per animal were examined at low (x10) and high (x20) magnification using an inverted Leica microscope equipped with a digital camera.

### Whole mount staining

Segments of distal ileum (4 cm long) were flushed with PBS, filled with fixative solution (4% neutral paraformaldehyde, 0.2% saturated picric acid in 0.1 M PBS), tied at both ends, and immersed in the same fixative solution. After 1 hour at 22°C, tissues were washed in PBS (3 x 10 min) and stored at 4°C until used. Under a dissecting microscope, a small incision was made with tweezers on a segment of ileum (approximately 1 cm long) and the longitudinal muscle layer with the adherent myenteric plexus (LMMP) was peeled off. The tissue sheets were gently stretched and pinned down on a wax support, washed twice with PBS, incubated in permeabilization buffer (0.5% Triton-X100 in PBS) then stained at room temperature for 16 hours with the appropriate rabbit primary anti- βIII-Tubulin (Sigma-Aldrich, Milan, Italy), anti-peripherin or anti-S100β (Millipore, Milan, Italy) antibodies (Table 1). Antibodies were diluted 1:400 in PBS/0.2% TritonX100/2% BSA [19]. TRITC-labeled secondary antibodies (Invitrogen, Milan, Italy; Table 1) were diluted 1:200 in PBS/0.2% TritonX100/2% BSA and incubated for 2 hours at room temperature to detect the immuno-complexes. Following extensive washing, in PBS/0.2% TritonX100 (3 x 10 min), samples were visualized with a Leica TCSNT/SP2 confocal microscope (original magnification x40). Microscope settings were established to collect images below saturation and were kept constant for all images.

**Table 1:** Primary and secondary antibodies used in the study.

**PRIMARY ANTIBODIES**			
**ANTIGEN (HOST)**	**CLONE**	**SOURCE**	**APPLICATION**
β-Actin (mouse)	AC-15	Sigma-Aldrich	WB
βIII-Tubulin (rabbit)	polyclonal	Couvance	WM
nNOS (rabbit)	polyclonal	Invitrogen	WB
Peripherin (rabbit)	polyclonal	Millipore	WM, WB
S100β (rabbit)	EP1576Y	Millipore	WM, WB
Substance P, SP (rabbit)	polyclonal	ImmunoStar	WM
**SECONDARY ANTIBODIES**			
**ANTIGEN (HOST)**	**CLONE**	**SOURCE**	**APPLICATION**
goat anti-rabbit IgG	HRP-conjugate	Sigma-Aldrich	WB
goat anti-rabbit IgG	Rhodamine-conjugate	Chemicon	WM
goat anti-mouse IgG	HRP-conjugate	Sigma-Aldrich	WB

WB, western blot; WM, whole mount.

### Immunoblot analysis

At the time of sacrifice, the small intestine was removed, washed in ice-cold PBS, cut into pieces 1 cm in length and placed over a sterile glass rod. The LMMP was quickly peeled off under a dissecting microscope (Leica). LMMP were homogenized in RIPA lysis buffer (150 mM NaCl, 50 mM Tris-HCl, 0.25% sodium deoxycholate, 0.1% Nonidet P-40, 100 μM NaVO_4_, 1 mM NaF, all provided by Sigma-Aldrich) containing protease inhibitors (0.5 mM EDTA, 0.1 mM PMSF, 1 μM leupeptin, 150 nM aprotinin). After 45 min at 4°C, particulate material was removed by centrifugation (15,000 xg for 30 min at 4°C) and protein concentration in the supernatants was determined using the bicinchoninic acid method (Pierce, Milan, Italy). Proteins were fractionated through an SDS-PAGE gel, immobilized onto a nitrocellulose membrane, and subsequently subjected to immunoblot analysis. The membranes were blocked for 1 hour with 5% non-fat dried milk in PBS with 0.05% Tween 20, then probed with the appropriate primary antibody (Table 1; 1:500 in PBS/0.05% Tween 20/5% non-fat dried milk for 16 hours at 4°C). Immuno-complexes were detected using horseradish peroxidase-conjugated secondary antibodies (Table 1; Sigma-Aldrich; 1:200 in PBS/0.05% Tween 20/5% non-fat dried milk for 2 hours at room temperature) and enhanced chemiluminescent system (Millipore). Images were captured using Hyper Film MP (GE Healthcare, Milan, Italy). Anti-mouse β-actin (Sigma-Aldrich) was used as the loading control. Densitometry analysis of the band intensity was performed using the ImageJ software (US National Institutes of Health).

### ELISA analysis

LMMPs were placed in ice-cold PBS (1:10 wt/vol) supplemented with protease inhibitors (0.1 mM PMSF, 1 μM leupeptin, 150 nM aprotinin) and homogenized for 30 seconds. Cellular debris was removed by centrifugation (10,000 xg for 10 min at 4°C). Levels of tumor necrosis factor (TNF)-α and interleukin (IL)-1β, IL-4, and IL-10 in the clear supernatants were determined by enzyme-linked immunosorbent assay (ELISA), using commercially available kits (eBioscience, Prodotti Gianni, Milano, Italy). The assays were conducted in accordance with the manufacturer’s recommended protocols.

### *In vivo* gastrointestinal transit

100 μL of fluorescein-isothiocyanate dextran (70,000 Da molecular weight; Santa Cruz Biotechnology, Heidelberg, Germany) dissolved in PBS (6.25 mg/mL) was administered via a orogastric tube into the stomach. Animals were placed in their original cages then sacrificed after 60 min by cervical dislocation. The abdomen was opened and the gut carefully removed avoiding stretching. Stomach and cecum were examined separately; small intestine and colon were divided into eight and two identical segments, respectively. Luminal contents were collected and clarified by centrifugation (10,000 xg, 15 min, 4°C). Fluorescence analysis was performed at 494/521 nm (Hitachi F2000; Hitachi, Tokyo, Japan). Gastrointestinal transit was calculated as the geometric center (GC) of distribution of the fluorescent probe using the formula:

GC=Σ(%oftotalfluorescentsignalpersegment×segmentnumber)/100.

### *Ex vivo* contractility studies

*Ex vivo* contractility studies were performed on distal 10 cm ileal segments. Neuronal-mediated contractions were evoked through electrical field stimulation (EFS; 2–40 Hz, 1-ms pulse-duration, 10-s pulse-trains, 60 V) using platinum electrodes connected to an S88 stimulator (Grass Instrument, Quincy, MA) or carbachol (Sigma-Aldrich) at concentrations ranging from 10^−9^ to 10^−4^ M.

### Bead latency test

As previously described [20], assessment of distal colonic transit and emptying, was performed by placing a spherical 2-mm glass bead 2 cm proximal to the anal opening using a fire-polished glass rod lightly coated with Surgilube lubricating jelly. After bead insertion, mice were placed in individual plastic cages lined with white paper to aid visualization of bead expulsion. The time required for expulsion of the glass bead was determined to the nearest 0.1 min for each animal.

### Fecal pellet expulsion and water content

To enumerate fecal pellets expulsed, each mouse was placed in a separate clean cage and observed for 60 minutes [21]. Fecal pellets were collected immediately after expulsion and placed in sealed 1.5 mL tubes. Tubes were weighed to obtain the wet weight of the stools, which were then dried overnight at 65°C and reweighed to obtain the dry weight. The stool water content was calculated from the difference between the wet and dry stool weights and expressed as a percentage.

### Statistical analysis

All results were given as mean ± standard error of the mean (SEM), except for distribution of the fluorescent probe, which was presented as median ± SEM. Differences in the mean for the different experimental groups was tested using one-way ANOVA analysis followed by Newman–Keuls or Bonferroni multicomparison *post hoc* tests. The levels of statistical significance are shown in figure legends. A *p*-value of 0.05 or less was considered statistical significant. Statistical analyses were performed using GraphPad Prism 3.03 software (GraphPad, San Diego, CA).

## Results

### Body weight and intestinal mucosa integrity

**Fig 2:**
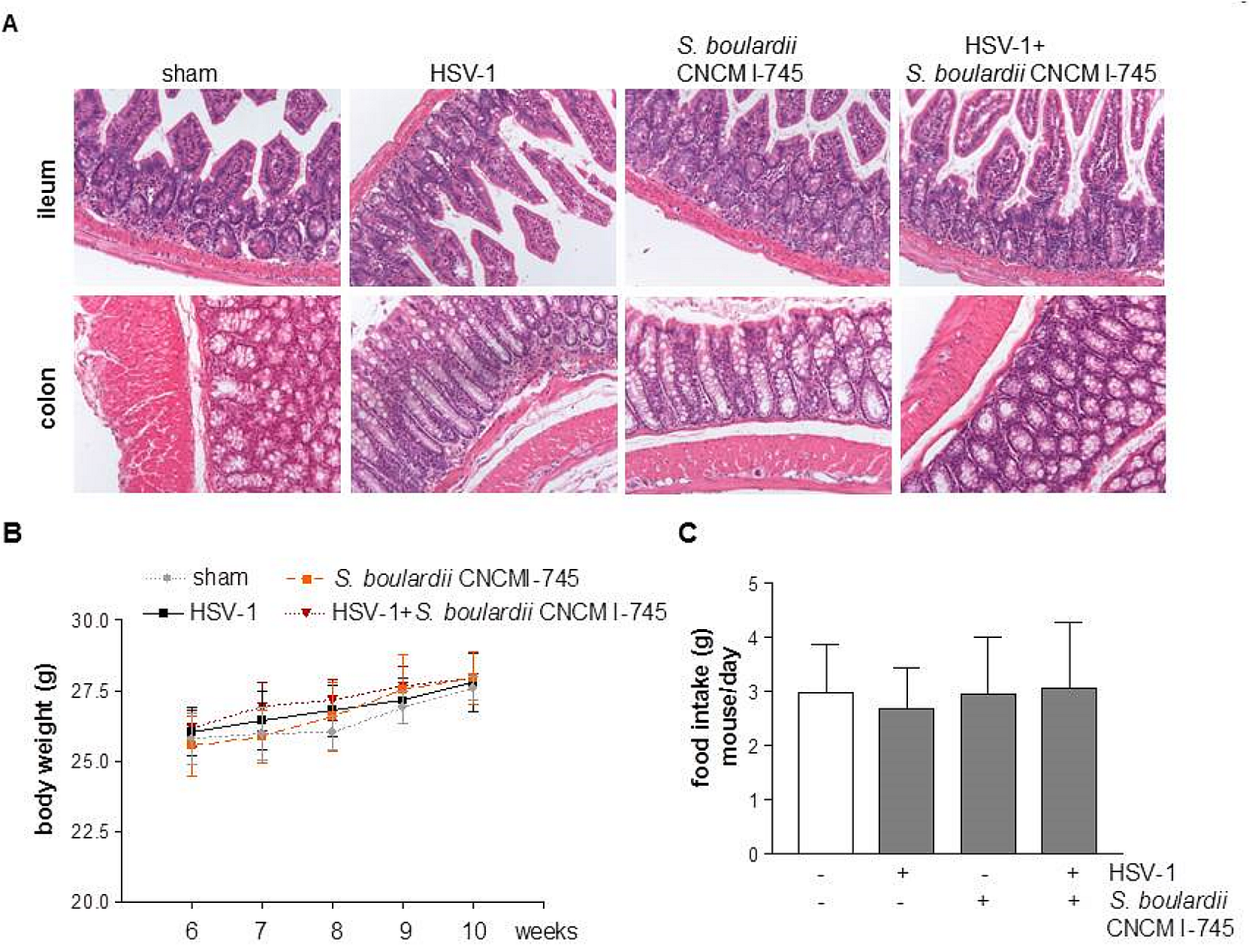
Effect of *S*. *boulardii* CNCM I-745 supplementation on mice. Panel A: Representative images of colons and ileum sections stained with hematoxylin/eosin. Panel B: body weights from 6 weeks after the IG inoculum. Panel C: daily food intake of mice from week 6 after IG inoculum. n = 8 mice per group. One way ANOVA and Bonferroni post-hoc test were employed to compare body weight and food intake. F(3,28) = 0.69, *p*>0.05.

Neurological deficit or sensorimotor dysfunction were not observed in HSV-1 infected mice throughout the experimental period. No histological anomalies in ileal and colonic mucosa were observed in any of the experimental groups (Fig 2A). Throughout the experiment, body weight gain and food intake were comparable between HSV-1 injected and control animals, either receiving *S*. *boulardii* CNCM I-745 or not (Fig 2B and 2C).

### HSV-1-induced gastrointestinal dysmotility

**Fig 3:**
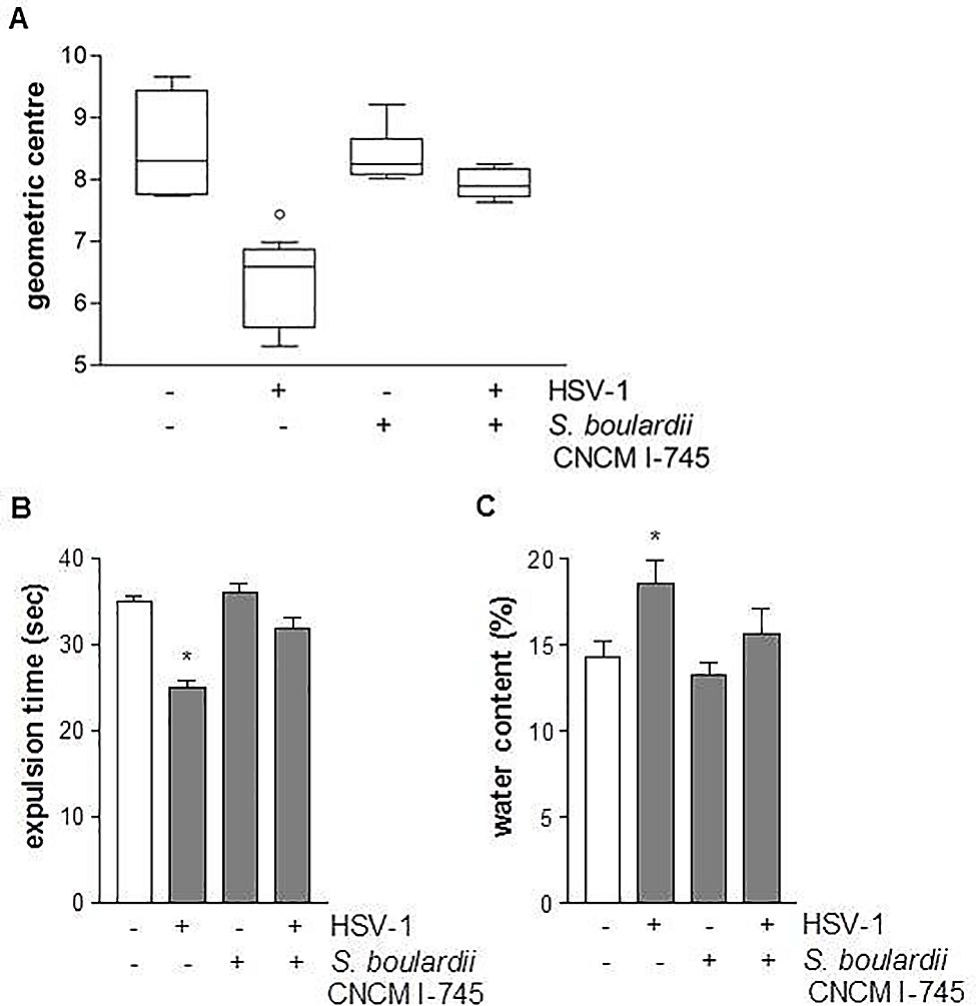
Effects of *S*. *boulardii* CNCM I-745 on gastrointestinal dysmotility induced by HSV-1 infection. Panel A: Relative distribution of non-absorbable FITC-labelled dextran probe in the intestine (geometric center, n = 8 per group). ° denotes *p*<0.02 versus control mice; Panel B: Expulsion time (seconds) for a glass bead inserted into the rectum. * denotes *p*<0.05 versus control mice; Panel C: Water content of the fecal pellets expulsed for 1 hour (n = 8 each group). * denotes *p*<0.05 versus control mice. For all the groups ANOVA gave an F value which is statistically significant with degrees of freedom.

Ten weeks after IG HSV-1 inoculum, mice experienced a loss in the coordinated motility of the different intestinal segments compared with non-infected mice (Fig 3). Thus, 10 weeks post viral inoculum a slower ileal transit (*p*<0.02 *vs* control mice) was associated with a quicker colonic emptying (*p*<0.05 *vs* control mice).

Daily supplementation with *S*. *boulardii* CNCM I-745 corrected the gastrointestinal dysmotility observed at 10 weeks post viral inoculum (Fig 3). Thus, *S*. *boulardii* supplementation normalized the ileal-colonic transit (*p*<0.02) and corrected the fecal water content and the bead expulsion time (*p*<0.05 *vs* HSV-1 infected mice, Fig 3).

### HSV-1-induced neuro-muscular dysfunction

**Fig 4:**
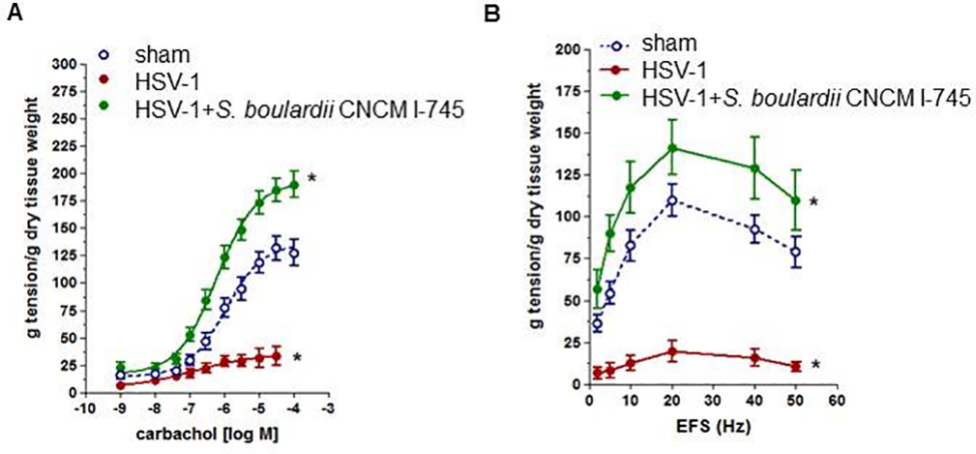
Effects of *S*. *boulardii* CNCM I-745 on ileal contractility impairment induced by HSV-1. Panel A: Carbachol-elicited contractions in ileum segments (n = 6–8 per group). Panel B: EFS-elicited contractions in ileum segments (n = 6−8 per group). One way ANOVA and Newman–Keuls *post hoc* test were employed to compare the extension of induced contractions between groups. * denotes *p*<0.01 versus control mice.

Since a coordinated gastrointestinal motility is largely dependent on the ENS, we assessed *ex vivo* the functional integrity of the myenteric plexus measuring the strength of pharmacologically (carbachol) and electrically induced muscular contractions of ileal segments. The strength of pharmacologically and electrically induced contractions of ileum segments isolated from HSV-1 infected mice was significantly reduced at week 10 post IG inoculum compared with controls (*p*<0.01, Fig 4). *S*. *boulardii* CNCM I-745 supplementation abrogated the HSV-1 induced deficit of the ileal neuromuscular unit (*p*<0.01 *vs* HSV-1 infected mice, Fig 4).

### HSV-1-induced anomalies of the ENS

The network of the ENS is usually mapped by evaluating specific neuronal and glial proteins, whose distribution and expression are erratic during gut inflammation and dysmotility. Fragmentation of intermediate neuronal filaments peripherin and βIII-tubulin, alteration of neuronal nitric oxide synthase (nNOS) levels, and under- or over-expression of the glial marker S100β are usually associated with intestinal neuropathology and motor dysfunction [9,22].

**Fig 5:**
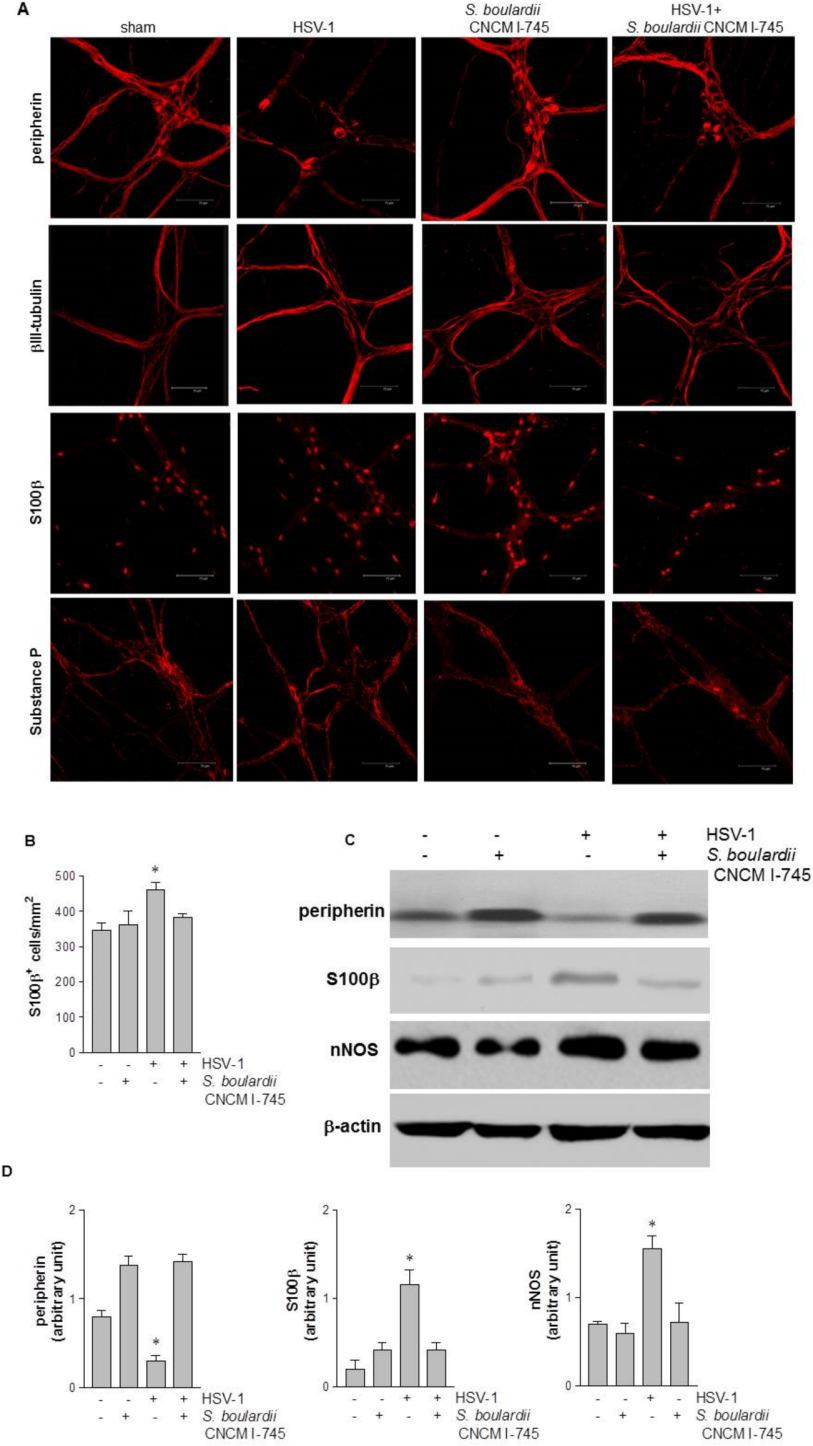
Effects of *S*. *boulardii* CNCM I-745 supplementation on HSV-1-induced neuroplasticity in the enteric nervous system. Panel A: Immunofluorescence analysis for neurofilament peripherin, neurotubules βIII-tubulin, glial marker S-100β, and neuropeptide substance P (SP). Scale bars 75 μm. Panel B: S100β positive cells per mm^2^ were quantified. Panel C: Western blot analysis of peripherin, S-100β, and nNOS in protein extracts from LMMP. β-actin was used as a loading control. Panel D: Protein amount was quantified by densitometry analysis. One way ANOVA and Bonferroni *post hoc* test were employed to compare number of S100β positive cells in Panel B and amounts of proteins in Panel D. In Panel B, * denotes F(3,28) = 10.88, *p*< 0.01 compared to control. In Panel D, ANOVA gave an F value which is statistically significant with degrees of freedom for all the groups; * denotes *p*< 0.05 compared to control.

To investigate the integrity of enteric nerves, in this study the distribution of peripherin and βIII-tubulin was evaluated by immunofluorescence staining. Ten weeks after IG administration of HSV-1, immunoreactivity of peripherin filaments was significantly reduced in non-supplemented mice compared with controls (Fig 5A, 5C and 5D). In contrast, the integrity of βIII-tubulin filaments was preserved even in presence of HSV-1 infection (Fig 5A). *S*. *boulardii* supplementation reduced HSV-1-mediated peripherin damage whereas βIII-tubulin filaments were unaffected (Fig 5A).

In addition to neuronal damage, IG HSV-1 inoculum induced an activated phenotype in enteric glial cells. Thus, the number of S100β^+^ cells in the LMMP of HSV-1 infected mice was increased compared with controls (*p*<0.01, Fig 5B). Moreover, S100β protein expression significantly augmented in LMMP of HSV-1 infected mice (*p*<0.05 *vs* control mice, Fig 5D). *S*. *boulardii* CNCM I-745 supplementation restored the expression of S100β at levels comparable with those in control mice (Fig 5).

Moreover, HSV-1 infection caused changes in the neurochemical code of LMMP neurons (Fig 5C and 5D); 10 weeks after HSV-1 exposure substance P (SP) positive nerve fibers were reduced while nNOS level was higher (*p*<0.05 vs control mice). *S*. *boulardii* CNCM I-745 supplementation moderated the neuroplastic changes in LMMP, preventing variations in expression and distribution of SP (Fig 5A) and nNOS (*p*<0.05 *vs* HSV-1 infected mice, Fig 5C and 5D).

### HSV-1-mediated inflammatory changes in the myenteric plexus

**Fig 6:**
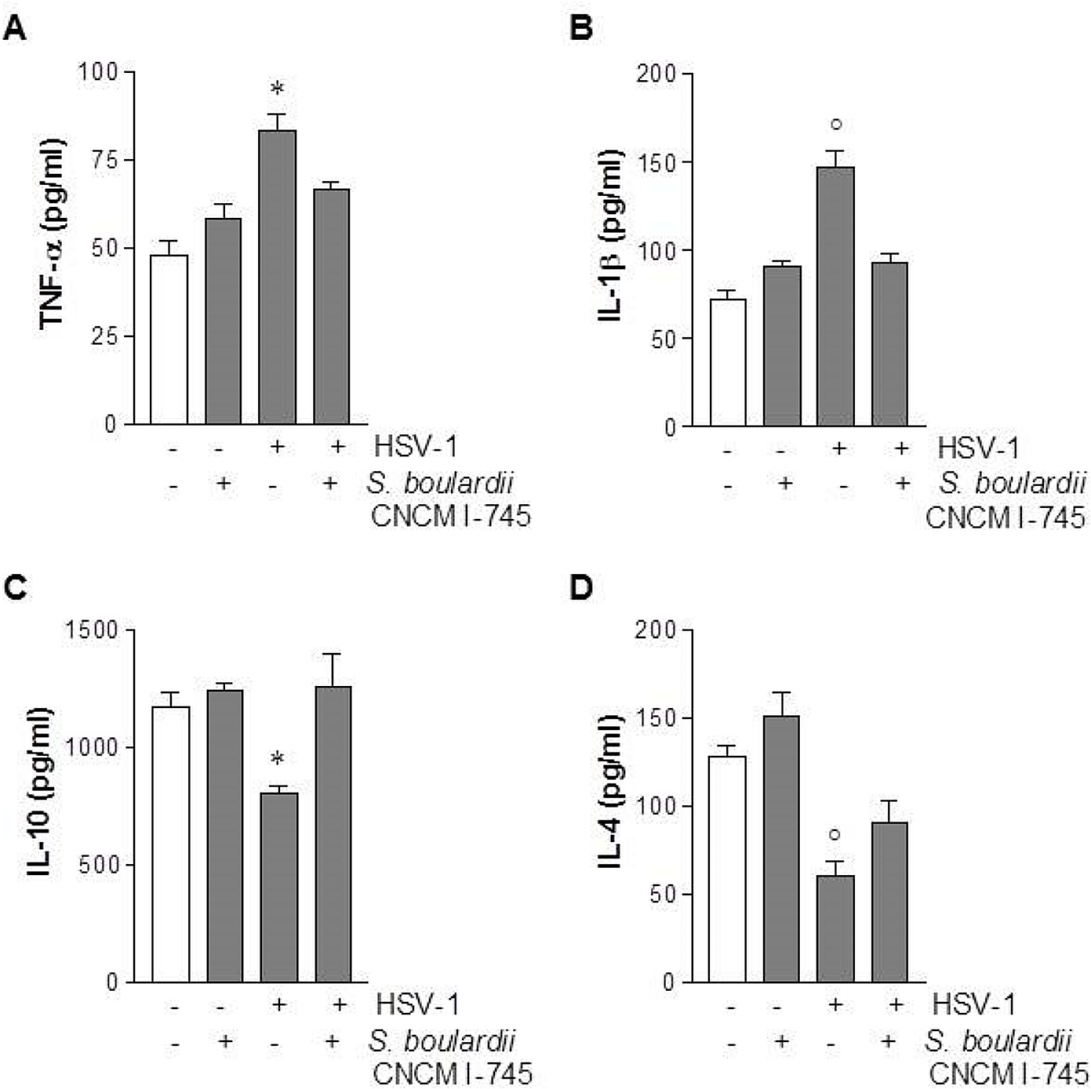
Effects of *S*. *boulardii* CNCM I-745 on HSV-1-induced inflammation in the longitudinal muscle layer with the adherent myenteric plexus (LMMP). TNF-α (Panel A), IL-1β (Panel B), IL-10 (Panel C), and IL-4 (panel D) quantified by ELISA. Cytokine levels were expressed as pg/mL of tissue lysate. One way ANOVA and Bonferroni *post hoc* test were employed to compare the levels of cytokines between groups. In Panel A, F(3,28) = 37.25, * denotes *p*<0.05 versus control mice; in Panel B, F(3,28) = 27.18, ° denotes *p* = 0.02 versus control mice; in Panel C, F(3,28) = 16.73, * denotes *p*<0.05 versus control mice; in Panel D, F(3,28) = 28.42, ° denotes *p* = 0.02 versus control mice.

To investigate whether the gastrointestinal dysmotility and the neuroplastic changes of the ENS were associated with an inflammatory process triggered by HSV-1 exposure, we evaluated the gut level of two relevant pro-inflammatory cytokines (TNF-α and IL-1β) using ELISA. Ten weeks after IG administration of HSV-1, intestinal levels of both TNF-α and IL-1β increased in non-supplemented HSV-1 infected mice compared with controls (*p*<0.05, TNF-α; *p*<0.02, IL-1β, Fig 6A and 6B). Indeed, in mice receiving *S*. *boulardii* CNCM I-745 supplementation, the HSV-1-induced surge in inflammatory cytokines was abolished (*p*<0.05, TNF-α; *p* = 0.02, IL-1β, Fig 6A and 6B). HSV-1 infection reduced levels of anti-inflammatory cytokines IL-10 and IL-4 (*p*<0.05 and *p*<0.02, respectively, compared with controls; Fig 6C and 6D). *S*. *boulardii* CNCM I-745 supplementation restored basal IL-4 and IL-10 levels in the gut of HSV-1 infected mice (*p*<0.05 for IL-10 and *p* = 0.02 for IL-4, Fig 6C and 6D).

## Discussion

By far the most common gastrointestinal motor disorder is IBS, affecting around 11% of the population globally, and often interfering with normal daily functioning [1,2,3]. Although three decades of extensive clinical research has attempted to identify a cure for IBS patients the results are undoubtedly unsatisfactory, mostly because of the complexity of the disease [2]. Moreover, the lack of reliable *in vivo* animal models mimicking the complexity of IBS symptoms has hampered the development of novel therapeutic strategies. [2,4]. In this study, we took advantage of a novel murine model of intestinal motor disorder, caused by a complex ENS dysfunction secondary to HSV-1 infection [9], to test the effect of the probiotic yeast *S*. *boulardii* CNCM I-745. We showed that *S*. *boulardii* CNCM I-745 supplementation effectively prevents gastrointestinal dysmotility and mucosal inflammation associated with the persistent HSV-1 infection of the ENS.

Gut dysmotility, microscopic inflammation, and visceral hypersensitivity are features present to varying extents in IBS patients, but are difficult to concurrently reproduce in animal models [23]. Typically, animal models utilize early-life pathological stressors, i.e., colonic irritation or maternal separation in newborn rats, to mimic at least some of the pathophysiological features of IBS such as the enhanced colonic motor function in response to stress [24,25]. Although currently there is no clinical evidence of an involvement of neurotropic viruses in IBS [2], our HSV-1 based model of intestinal dysmotility offers some unique features relevant to the IBS field, such as the presence of mild intestinal inflammation combined to structural and functional anomalies of the enteric nerves [9]. Indeed, several studies have demonstrated that IBS patients show a low-grade inflammation throughout the small bowel and colon therefore affecting nearby enteric nerves through the release of inflammatory mediators, including interleukins and histamine [7,26,27]. It has also been suggested that enteric nerves in IBS patients undertake significant phenotypic and molecular neuroplasticity eventually accounting for symptoms and pathophysiology [28,29]. Indeed, in our model HSV-1 triggered significant neuroplastic changes in the ENS (Fig 5) including the fragmentation of intermediate neuronal filaments markers of intestinal neuropathology associated with gut motor dysfunction [9,22]. Structural and functional alterations in enteric glia cells, such as the increase in S100β, have been described in animal models of stress-associated gastrointestinal disorders [30] as well as in IBS patients [31]. These observations suggest an abnormal activation of EGC-enteric nerve unit contributing to the visceral hypersensitivity in these patients. Moreover, following HSV-1 IG inoculum we observed an increase in SP positive fibers, as has been reported in colonic mucosa of IBS patients and whose activity correlates with visceral pain [32,33]. Overall, the changes induced by HSV-1 in the gut mimic most of the anomalies observed in the ENS of IBS patients, and therefore may represent a dependable animal model to study novel therapeutic interventions.

Here we reported that administration of *S*. *boulardii* CNCM I-745 mitigated the HSV-1 induced alteration of the ENS, restored the gut neuromuscular function (Fig 4), and colonic stool water resorption (Fig 3). Our data are consistent with available clinical studies reporting favorable effects of *S*. *boulardii* CNCM I-745 supplementation on the gastrointestinal functional anomalies observed in IBS patients [16,34,35,36]. In 2008, Swidsinski *et al*. also concluded that *S*. *boulardii* significantly improved the fecal biostructure and normalized diarrheal symptoms in patients with chronic idiopathic diarrhea [37]. On the other hand, Bafutto and colleagues reported that *S*. *boulardii* significantly improved GI symptoms in IBS patients and Abbas and colleagues demonstrated an improvement of quality of live and a significant reduction in mucosal inflammatory cytokines [34,36]. Interestingly, recent studies reported the effectiveness of other probiotic yeasts in IBS patients. In a randomized clinical trial on IBS-diarrhea type, Pineton de Chambrun *et al*. showed that *S*. *cerevisiae* CNCM I-3856 reduced abdominal pain and discomfort scores. However, this probiotic failed to improve tool frequency and consistency [38].

Probiotic-based treatments are gaining popularity for the treatment of multiple gastrointestinal disorders including IBS [39,40]. A number of hypotheses have been proposed to justify their use, such as beneficial effects on immune homeostasis of the host intestinal mucosa, production of short-chain fatty acids which alter gut motility, and the manipulation of opioid receptors [41,42,43]. However, the quality of study design, the heterogeneity of treatment protocols, and variability of treatment (single strains versus mixtures) makes it difficult to demonstrate the efficacy of probiotic administration in IBS [10,44]. Since studies performed using intestinal biopsies support the existence of an inflammatory milieu in IBS patients, the effectiveness of specific probiotics may depend on their anti-inflammatory effects [39]. For example, *Bifidobacterium infantis* 35624 demonstrated reproducible anti-inflammatory effects both in animal models [45] and *in vitro* and showed efficacy in IBS patients [12,46]. Here we report that *S*. *boulardii* CNCM I-745 administration attenuated HSV-1 induced immune-mediated inflammatory process and had effects on mucosa and muscular layers of both the small intestine and colon (Figs 3, 4 and 6). Several studies have proven the ability of this yeast to modulate mucosal inflammation by blunting the activity of nuclear factor κB [47] and mitogen-activated protein kinases ERK1/2 [48], or by enhancing the expression of peroxisome proliferator-activated receptor-gamma [49]. In addition, the ability of *S*. *boulardii* CNCM I-745 to trap activated T cells in mesenteric lymph nodes could reduce intestinal inflammation and safeguard the integrity of the ENS [50]. Indeed, *S*. *boulardii* interaction with resident microflora and intestinal mucosa modulates the intraluminal inflammatory cytokines leading to an improvement in mucosal barrier function or normalization of the activity of the ENS, both accounting for the amelioration in intestinal motility as it has been suggested for other probiotics [12]. Moreover, *S*. *boulardii* treatment significantly decreased blood and tissue levels of proinflammatory cytokines and increased anti-inflammatory IL-10 level with a marked improvement in the quality of life and bowel-related symptoms [34]. Indeed, this study was designed to investigate the mechanisms of actions of *S*. *boulardii* CNCM I-745 in the light of previously described improvement in quality of life of IBS patients [16,34,51]. Of course, the complexity of IBS etiological factors cannot be reflected in a single animal model. We are aware of the limitations of our experimental model and further investigations of IBS symptoms treatment by *S*. *boulardii* could take advantage by different *in vivo* models [52,53].

Overall the results of our study indicate that administration of *S*. *boulardii* CNCM I-745 ameliorates gastrointestinal dysmotility that follows HSV-1 IG administration. HSV-1-induced dysmotility is associated with mild inflammation, thus partially resembling the pathophysiological picture of IBS patients. Similar to other bacterial probiotics effective on IBS symptoms, the well-known anti-inflammatory and immuno-modulatory properties of *S*. *boulardii* CNCM I-745 [54] appear to be essential for the beneficial effects in our model of gastrointestinal dysmotility. In addition, the observations that *S*. *boulardii* CNCM I-745 prevents gut dysbiosis even during antibiotic administration [55,56], might suggest additional mechanisms of action.
