# Correction: *Saccharomyces boulardii* CNCM I-745 supplementation reduces gastrointestinal dysfunction in an animal model of IBS

**DOI:** 10.1371/journal.pone.0188563

**Published:** 2017-11-17

**Authors:** Paola Brun, Melania Scarpa, Chiara Marchiori, Gloria Sarasin, Valentina Caputi, Andrea Porzionato, Maria Cecilia Giron, Giorgio Palù, Ignazio Castagliuolo

The affiliation for the second author is incorrect. Melania Scarpa is not affiliated with #1 but with Veneto Institute of Oncology IOV–IRCCS, Padova, Italy.
